# Case Report: A Novel Pathomechanism in PEComa by the Loss of Heterozygosity of *TP53*

**DOI:** 10.3389/fonc.2022.849004

**Published:** 2022-03-28

**Authors:** Henriett Butz, József Lövey, Márton Szentkereszty, Anikó Bozsik, Erika Tóth, Attila Patócs

**Affiliations:** ^1^ Department of Molecular Genetics, National Institute of Oncology, Budapest, Hungary; ^2^ Hereditary Tumours Research Group, Hungarian Academy of Sciences-Semmelweis University, Budapest, Hungary; ^3^ Department of Radiotherapy, National Institute of Oncology, Budapest, Hungary; ^4^ Department of Oncology, Faculty of Medicine, Semmelweis University, Budapest, Hungary; ^5^ Surgical and Molecular Tumor Pathology Centre, National Institute of Oncology, Budapest, Hungary

**Keywords:** Li–Fraumeni syndrome, heritable *TP53*-related cancer syndrome, *TP53*, p53, PEComa, germline mutation, Li-Fraumeni, Li-Fraumeni spectrum

## Abstract

Since the introduction of next-generation sequencing, the frequency of germline pathogenic *TP53* variants and the number of cases with unusual clinical presentations have been increasing. This has led to the expansion of the classical Li–Fraumeni syndrome concept to a wider cancer predisposition syndrome designated as the Li–Fraumeni spectrum. Here, we present a case with a malignant, metastatic perivascular epithelioid cell tumor (PEComa) of the thigh muscle and a sinonasal carcinoma harboring a novel *TP53* germline splice mutation (NM_000546.5:c.97-2A>C). The classical presentation of LFS in the long-since deceased mother and the presence of a germline *TP53* variant in the proband suggested a possible familial *TP53-*related condition. Complex pathological, molecular, and clinical genetic analyses (whole exome sequencing of germline variants, multigene panel sequencing of tumor DNA, Sanger validation, an *in vitro* functional test on splicing effect, 3D protein modeling, p53 immunohistochemistry, and pedigree analysis) were performed. The *in vitro* characterization of the splice mutation supported the pathogenic effect that resulted in exon skipping. A locus-specific loss of heterozygosity in the PEComa but not in the sinonasal carcinoma was identified, suggesting the causative role of the splice mutation in the PEComa pathogenesis, because we excluded known pathogenetic pathways characteristic to PEComas (*TSC1/2*, *TFE3*, *RAD51B*). However, the second hit affecting *TP53* in the molecular pathogenesis of the sinonasal carcinoma was not identified. Although PEComa has been reported previously in two patients with Li–Fraumeni syndrome, to the best of our knowledge, this is the first report suggesting a relationship between the aberrant *TP53* variant and PEComa.

## Introduction

Germline pathogenic *TP53* variants are associated with Li–Fraumeni syndrome (LFS), which is a rare, autosomal-dominant, hereditary tumor syndrome (1). Five cancer types account for the majority of LFS tumors, which are called “LFS core” tumors: adrenocortical carcinoma, breast cancer, central nervous system tumors, osteosarcomas, and soft-tissue sarcomas. However, LFS patients have an increased risk of several additional cancers, such as leukemia, lymphoma, gastrointestinal cancers, and cancers of head and neck, kidney, larynx, lung, skin, ovary, pancreas, prostate, testis, and thyroid (2). Also, many cases of germline *TP53* pathogenic variants have been identified in children with cancers, or among adult females with breast cancers without a familial history of cancer. Hence the expansion of the LFS concept to a wider cancer predisposition syndrome: the terms “heritable *TP53*-related cancer (hTP53rc) syndrome” by the European Reference Network GENTURIS and “Li-Fraumeni spectrum” by Kratz et al. have been recently suggested (3, 4). Based on classic, familial cases, the cumulative cancer risk was initially given as 73%–100% by age 70, with risks close to 100% in women (5–7). However, based on population studies, and as a consequence of the increased availability for high-throughput testing, the overall cancer penetrance seems to be lower (3, 8). Still, based on a recent observational cohort study on cancer incidence, patterns, and genotype–phenotype associations, individuals with Li–Fraumeni syndrome had a nearly 24 times higher incidence of any cancer than the general population (9). Additionally, while the disease prevalence is not well established, the prevalence of the germline pathogenic *TP53* carrier status in the general population was recently estimated to be approximately 1:4,500 (8).

In clinical genetics, testing criteria for the *TP53* gene have been extensively discussed (3, 4), and for most tumors, based on the personal or family history suggestive of such a syndrome, germline testing is recommended (10). In addition, *TP53* pathogenic/likely pathogenic (P/LP) variants are commonly detected somatically, and it is the most frequently mutated gene in tumor tissues (11–13). Therefore, it has been recently recommended that when only somatic testing is performed and a P/LP variant is identified in the *TP53* gene, germline examination is indicated only when it is detected in sarcomas, breast cancer, or brain tumors (10).

In this current study, we report a peculiar case, where in the background of an unusual appearance of the Li–Fraumeni spectrum manifesting in a malignant perivascular epithelioid cell tumor (PEComa), a novel *TP53* pathogenic variant was identified. While PEComa was described in two previous case reports of Li–Fraumeni patients (14, 15), PEComa was the first manifestation of the disease in our patient. Our molecular genetic assays suggest a potential relationship between the pathogenic *TP53* variant and PEComa development.

## Methods

### Immunohistochemistry

Immunohistochemical characterization was performed on a Ventana Benchmark autostainer (Roche Tissue Diagnostics, Oro Valley, AZ, USA) using the ultraView Universal DAB Detection Kit. Antibodies used (in alphabetical order) and vendors were as follows: ERG, H-Caldesmon, MelanA, and SOX10 (Ventana, Oro Valley, AZ, USA). Further antibodies CD34 (Dako-Agilent, Santa Clara, CA, USA, 1:200), Desmin (Dako-Agilent, 1:200), EMA (Dako-Agilent, 1:800), H3K27me3 (Cell Signaling, Danvers, MA, USA, 1:50), HHF35 (Dako-Agilent, 1:50), HMB45 (Dako-Agilent, 1:50), S100 (Dako-Agilent, 1:4000), SMA (Dako-Agilent, 1:100), STAT6 (Santa Cruz, Dallas, TX, USA, 1:100), Vim (Dako-Agilent, 1:100), and p53 (Dako-Agilent, 1:200) were used.

### Fluorescent *In Situ* Hybridization

Fluorescent *in situ* hybridization (FISH) was performed using the ZytoLight^®^ SPEC EWSR1/FLI1 TriCheck™ and ZytoLight^®^ SPEC TFE3 Dual Color Break Apart Probe.

### Genetic Analysis

Germline genetic analysis of the proband and family members was performed following an informed consent based on the ethical approval by the Scientific and Research Committee of the Medical Research Council of the Ministry of Health, Hungary (ETT-TUKEB 53720-4/2019/EÜIG).

#### Nucleic Acid Isolation From Peripheral Blood and From Tumor Tissue

DNA purification from peripheral blood and formalin-fixed paraffin-embedded (FFPE) tissues was performed using the Gentra Puregene Blood Kit (Cat No.: 158389, Qiagen, Hilden, Germany) and the Maxwell RSC DNA FFPE Kit on a Maxwell RSC Instrument (Cat. No.: S1450, Madison, WI, USA) as part of the routine molecular pathology diagnostic workflow. For RNA analysis, blood was collected in Tempus™ Blood RNA Tubes (Thermo Fisher Scientific, Waltham, MA, USA) and RNA extraction was performed by using the Tempus™ Spin RNA Isolation Kit. Nucleic acid quality and quantity were determined by a NanoDrop^®^ 1000 Spectrophotometer (NanoDrop Technologies, Thermo Fisher Scientific, Waltham, MA, USA).

#### Whole Exome Sequencing From Peripheral Blood

Whole exome sequencing was done as previously reported, using a Twist Human Core Exome library preparation with a Twist mitochondrial panel (Cat. No.: 102026, Twist Bioscience, San Francisco, CA, USA) on a NovaSeq Illumina platform (Illumina, San Diego, CA, USA) with an average coverage of 100x (16). Data were analyzed by applying the Genome Analysis Toolkit (GATK) Germline short variant discovery (SNPs + Indels) algorithm. Annotation of coding variants was performed, following the American College of Medical Genetics and Genomics (ACMG) recommendations (17).

#### Sanger Validation, Site-Specific LOH Analysis, and RNA Splicing Effect Test

Sanger validation and site-specific LOH analysis were performed as previously reported (16). Primers used for validation were as follows: TP53_ex04_FOR 5′-CTGGTAAGGACAAGGGTTGG-3′; TP53_ex04_REV: 5′-GCCAGGCATTGAAGTCTCAT-3′, and for LOH testing: TP53_int4ex4_F1: 5′-CTGGTAAGGACAAGGGTTGG-3′; TP53_int4ex4_F2: 5′-ACTTCCTGAAAACAACGTTCTG-3′; TP53_int4ex4_R1: 5′-TCATCTGGACCTGGGTCTTC-3′; TP53_int4ex4_R2: 5′-TCTGGACCTGGGTCTTCAGT-3′; TP53_int4ex4_R3: 5′-TCTGGGAGCTTCATCTGGAC-3′.

For testing the splicing effect, RNA extracted from whole blood was reverse transcribed using SuperScript IV Reverse Transcriptase (Thermo Fisher Scientific, MA, USA). cDNA was then PCR-amplified with the following primers: TP53-C-e02_For: 5′-AGGAAACATTTTCAGACCTATGGA-3′, TP53-C-e06_Rev: 5′-CTGTCATCCAAATACTCCACACG-3′. PCR products were subjected to agarose gel electrophoresis next to controls and were then submitted for Sanger sequencing.

#### Multigene Panel Sequencing on FFPE Tumor DNA

Multigene panel sequencing of 161 genes related to personalized tumor therapy with Oncomine™ Comprehensive Assay v3M (Cat. No.: A35805, Thermo Fisher Scientific, Waltham, MA, USA) was performed as previously described on an Ion Torrent next-generation sequencing platform (Ion GeneStudio S5 System, Thermo Fisher Scientific, Waltham, MA, USA) (16). Data were analyzed using Oncomine Knowledge Reporter Software (Cat. No.: A34298, Thermo Fisher Scientific, Waltham, MA, USA).

#### Monogenic Mutation Analysis of Sinonasal Carcinoma

A real-time PCR test, a cobas 4800 KRAS Mutation test, and a *BRAF/NRAS* mutation test were used according to the manufacturer’s instructions.

#### 3D Protein Modeling

For protein modeling, prediction, and analysis, Phyre2 software was used to compare wild-type and variant amino acid sequences (18). For assessing the variant protein function and disorder prediction, the Phyre Investigator algorithm was applied.

#### Variant Classification

Specifications of the ACMG/AMP variant interpretation guidelines for germline *TP53* variants by Fortuno et al. were applied for variant classification (19). Accordingly, the ClinGen Sequence Variant Interpretation (SVI) Committee-approved decision tree (Abou Tayoun et al.) was used to determine the strength of PVS1 criteria, similarly to the *TP53*(NM_000546.5):c.97-1G>A variant (19, 20).

## Results

### Case Report

A 90 × 60 × 115 mm soft tissue tumor was observed in the medial part of the right thigh of a 38-year-old, Caucasian male patient. In addition to the right-thigh tumor, soft-tissue MRI and thoraco-abdominal and pelvic CT revealed three nodules in the chest that appeared suspicious for metastatic processes (15-mm nodule in the right-lobe S10; 102-mm nodule in the left-side S6 segment and a 10 × 15 mm nodule subcarinal). Following the surgical removal of the thigh soft-tissue tumor, which was diagnosed as a grade III myxofibrosarcoma, chemotherapy (6 series of EPI-ADM, parallel Lartruvo treatment from the second series) was started (**Table 1**).

**Table 1:** Timeline of the patient history.

Date	Event
25 June 2018	Ultrasound confirmation of a lump on the right thigh (90 × 60 × 115 mm inhomogeneous, vascularized, cystic lesion)
3 July 2018	MRI of the thigh identified a 87 × 79 × 120 mm lesion
5 July 2018	Chest, abdominal, pelvic CT for staging identified 3 lesions suspected as metastasis in the lung (right lobe S10 segment -15 mm, S6 - 102 mm, subcarinal 10 × 15 mm)
11 July 2018	Surgical removal of the thigh lesion. Histological diagnosis: myxofibrosarcoma grade III. Following surgery, chemotherapy was started (6 cycles epiADM, from the second cycle with additional Latruvo treatment)
5 October 2018	Control MRI of the thigh: no tumor/recurrence was found
15 November 2018	Chest, abdominal, pelvic CT: lung nodules were regressed (right lobe S10 segment -12 mm, S6 - 6 mm, subcarinal 11 × 7 mm).
4 December 2018	Consultation of thoracic surgery: radiation therapy of the lung and mediastinal nodules are recommended. Right S10 and subcarinal nodules can be removed by minimal invasive approach.
20 January 2019	Video-assisted thoracoscopic surgery, VATS
21 February 2019	Histology of the lung nodules and lymph nodes: No malignancy can be detected. Necrotizing granulomatous inflammation.
3 March 2020	The patient observed nasal congestion in the right nostril along with bloody rhinorrhea
5 May 2020	Endoscopic Surgery (following head and face CT & MRI), histology: adenocarcinoma, intestinal type
9 June 2020–16 July 2020	Radiochemoterapy (tumor bed irradiation with 54 Gy, along with cisplatin and 5FU chemotherapy)
9 September 2020	Control MRI of the skull and neck; CT of the skull and rhinobasis. Postoperative radiotherapy resulted in complete regression of the sinonasal lesion. No residual or recurrent tumor can be detected
4 October 2020	Control chest CT scan revealed a 27-mm nodule in the S10 mediastinal segment of the right lobe. Consultation for thoracic surgery recommended removal.
29 October 2020	PET/CT scan identified FDG uptake in a soliter nodule in the right lower lobe nodule, suggesting a metastasis in the lung.
16 November 2020	Thoracic surgery: right lobectomy
27 November 2020	Histology: I. metastasis of a malignant PEComa (grade III) in the right lobe; II: lymph nodes are tumor free
14 December 2020	Control MRI of the skull and neck; CT of the skull and rhinobasis: no residual or recurrent tumor can be detected
7 January 2021	Tumor board recommended close follow up
7 April 2021	Control MRI of the skull and neck; CT of the skull and rhinobasis: no residual or recurrent tumor can be detected
17 June 2021–14 July 2021	Control whole body MRI (abdomen, pelvis and thigh), and skull and neck MRI and spine & chest MRI: no residual or recurrent tumor can be detected
17 November 2020–24 November 2020	Control MRI of the skull, chest, abdomen, and thighs indicated no residual or recurrent tumor

As the pulmonary nodules moderately regressed following chemotherapy, pulmonary surgery was performed to remove residual right-lobe nodules. Histology showed necrotizing granulomatous inflammation.

Three months later, the patient observed bloody rhinorrhea. Upon CT scanning, soft-tissue densities were observed in the sinonasal tract. After endoscopic surgery, intestinal-type sinonasal carcinoma showing typical histology and immunophenotype was diagnosed. Postoperative radiotherapy resulted in complete regression of the sinonasal tumor.

**Figure 1:**
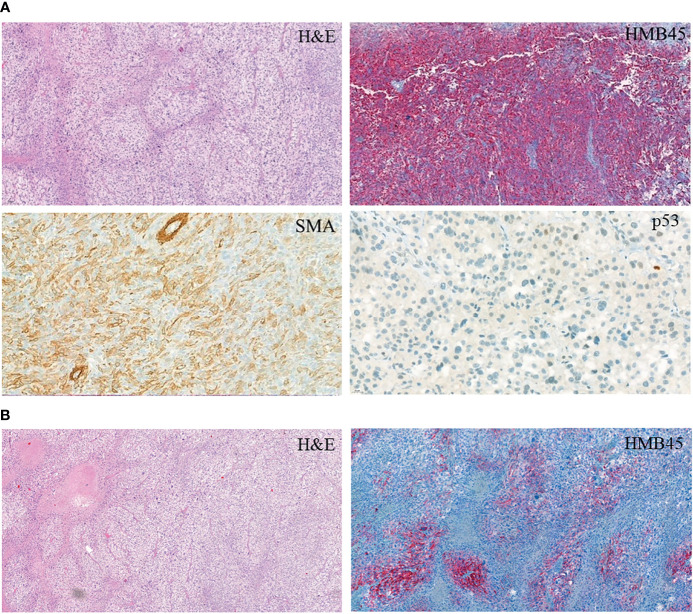
Immunohistochemistry of the PEComa tissue. **(A)** PEComa metastasis in the lung. **(B)** Primary tumor.

Ten months later, a control examination showed a right-lung nodule: therefore, a right lower lobectomy was performed. Pathological investigation revealed a cellular tumor showing a prominent perivascular arrangement. Tumor cells were pleomorphic with epithelioid or spindle-shaped character, and they had clear or abundant granular eosinophilic cytoplasm. Extensive necrotic areas and a very high mitotic rate (77/10HPF) were observed (**Figure 1**). Upon immunohistochemistry, tumor cells showed diffuse vimentin positivity. In the clear cell areas, tumor cells showed diffuse HMB45 positivity (**Figure 1A**). Focal but strong HMB45, desmin, H-Caldesmon, and smooth-muscle-actin expression were seen in the spindle cell areas (**Figure 1A**). Labeling for S100, SOX10, MelanA, cytokeratin (AE1–AE3), EMA, HHF35, CD34, STAT6, H3K27me3, and ERG was negative. Tumor cells were almost completely negative for p53 immunohistochemistry. Only scattered pleomorphic cells showed weak p53 expression. *EWSR1* and *TFE3* fluorescent *in situ* hybridization showed no rearrangement of the examined genes. The final diagnosis was metastasis of a malignant PEComa (grade III). In light of the histopathological results of the pulmonary lesion, the histological findings of the thigh tumor, which was originally diagnosed as a myxofibrosarcoma by another institute, were reevaluated by a specialist soft-tissue pathologist. Although the thigh tumor showed focal myxoid areas, probably resulting in the original diagnosis of myxofibrosarcoma, morphologically it was a similar mixture of epithelioid and spindle cells, as seen in the pulmonary lesion. Since only a limited panel of immunohistochemistry was performed at the time of the primary diagnosis, further immunohistochemistry including muscle markers and HMB45 was performed, which showed the same positive reaction as in the lung tumor (**Figure 1B**). As the thigh tumor showed a similar morphology and immunophenotype, it was reclassified as a primary PEComa, and the pulmonary tumor was considered as its metastasis.

Following lobectomy, a control CT was negative. Twelve months after the lung surgery, the patient appeared to be tumor free and is under close clinical follow-up (**Table 1**).

### Molecular and Clinical Genetic Findings

We had only a limited amount of tissue from the sinonasal carcinoma, which was unfortunately not sufficient for multigene analysis. Monogenic analysis using COBAS kits showed no evidence of *KRAS*, *NRAS*, or *BRAF* mutation.

**Figure 2:**
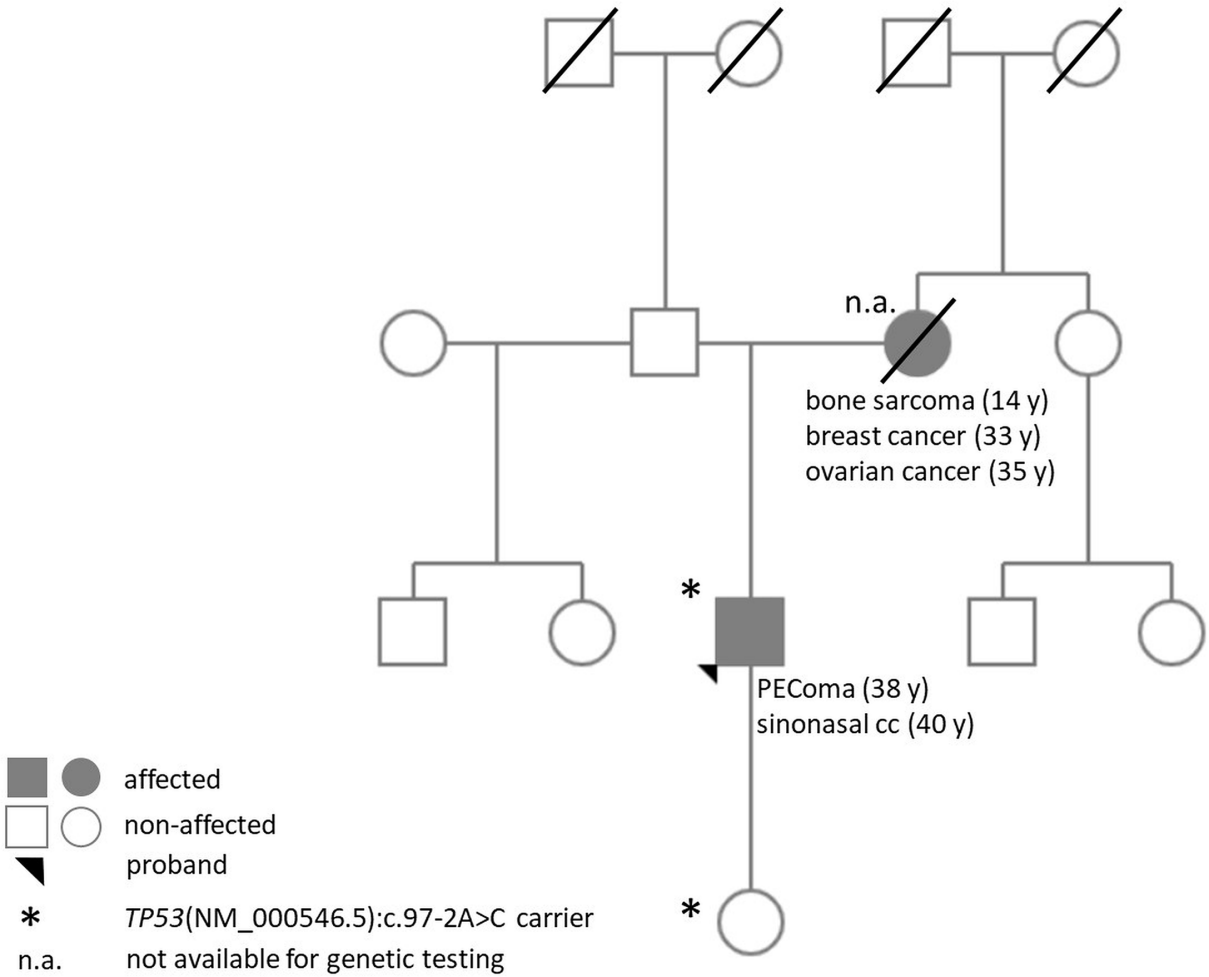
Pedigree of the proband.

Multigene panel (161 genes) sequencing was performed in the malignant PEComa and identified a *TP53*(NM_000546.5):c.97-2A>C variant with 66.21% allele frequency (variant allele frequency, VAF), but no other therapy-predictive pathogenic variant or gene fusion was detected. As VAF of the *TP53* variant suggested a potential germline presence, the patient was referred for genetic consultation and molecular genetic analysis in our department. During the consultation and pedigree analysis, Li–Fraumeni core tumors in the long-since deceased mother of the proband were identified (osteosarcoma at age 14; breast cancer at the age of 33 and ovarian cancer at the age of 35). Based on the available information, no other relative was affected (**Figure 2**).

**Figure 3:**
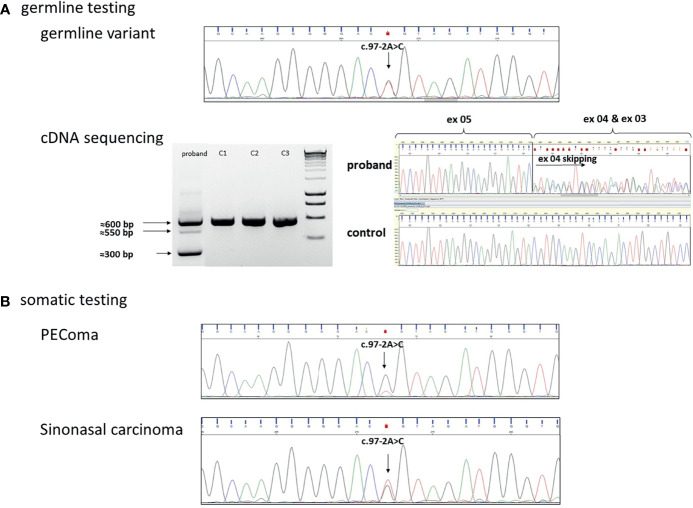
**(A)** Germline heterozygous *TP53*(NM_000546.5):c.97-2A>C variant in the DNA isolated from blood. cDNA sequencing identified exon 4 skipping, accordingly. On the electrophoresis gel, the 600-bp PCR product indicates the wild type, the 550-bp PCR product indicates a heteroduplex, and the 300-bp PCR product was confirmed as a skipped exon 4 transcript following Sanger sequencing. (C1, C2, C3 were used as controls). **(B)** Sanger sequencing in the PEComa tissue sample indicated the loss of the wild-type (wt) allele in the tumor: loss of heterozygosity (LOH) was detected. In sinonasal carcinoma, wild-type and variant alleles are presented in ~50%–50%: no LOH was detected.

We performed targeted Sanger sequencing of the identified variant and proved the germline presence of the *TP53*(NM_000546.5):c.97-2A>C variant (**Figure 3A**). Exome sequencing was also performed but was not able to identify other pathogenic variants in the compulsory gene list report or in potential hereditary cancer genes. Comparing the germline and the somatic (tumor types) variants, a partial locus-specific loss of heterozygosity (normalized reduction of the reference allele quantity was 0.3 relative to the variant allele) was observed in the PEComa, whereas no LOH was identified in the sinonasal carcinoma (**Figures 3A, B** **)**. Additionally, *in vitro* RNA testing proved whole exon 4 skipping due to the *TP53*(NM_000546.5):c.97-2A>C variant (**Figure 3A**).

**Figure 4:**
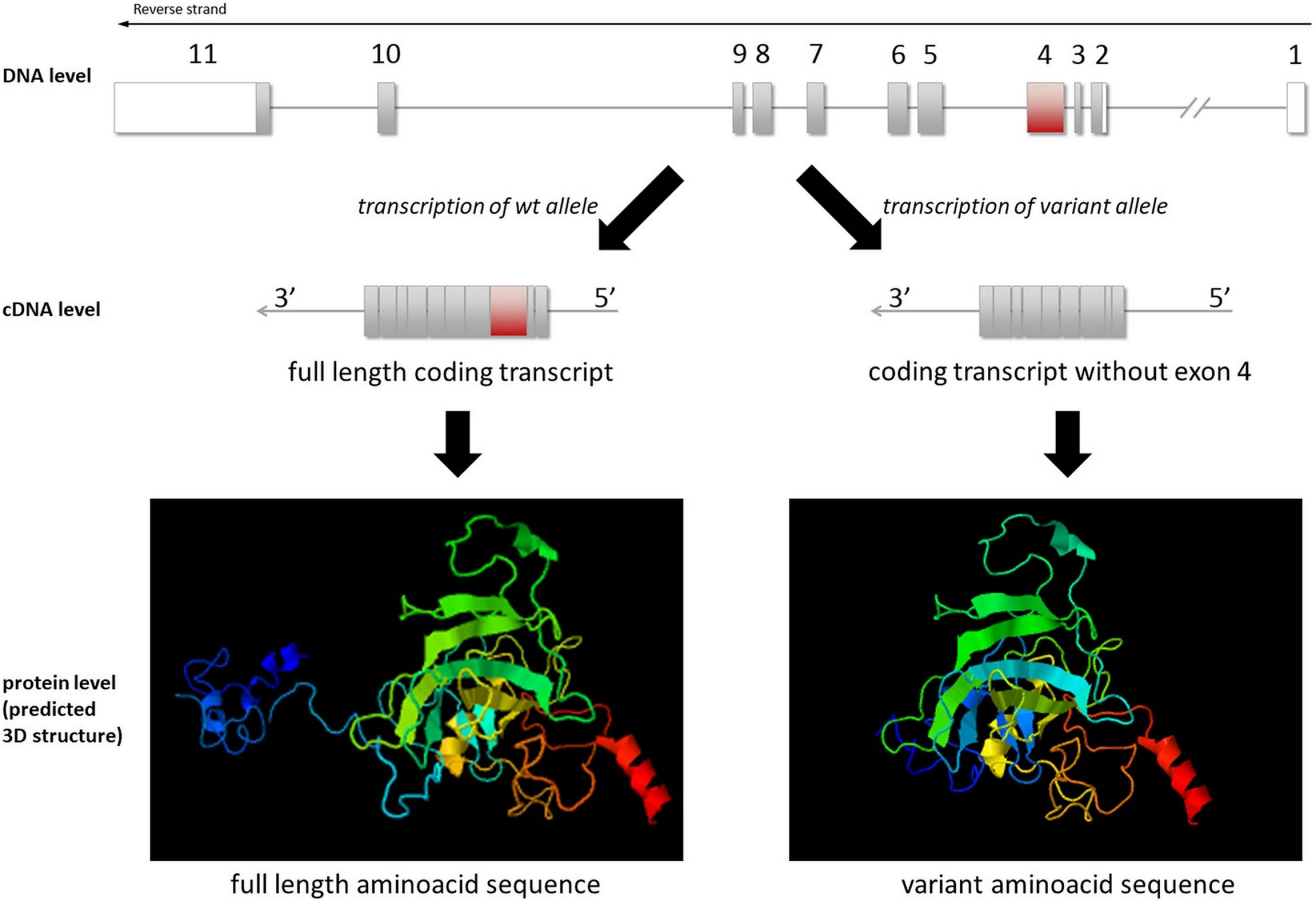
Illustration of *TP53*(NM_000546.5) exon 4 skipping at the DNA, cDNA, and protein levels. 3D modeling of the wild-type and variant protein indicated different structures (visualized by JSmol).

While this exon skipping does not lead to a frame shift, it results in a loss of 93 amino acids (from amino acids 32 to 125) at the protein level. Based on protein modeling, the variant protein was predicted to have a different 3D structure (**Figure 4**). Additionally, 40.8% of the lost amino acids were predicted as “disordered” in the 3D structure, meaning that the change or loss might lead to damaged protein structure/function. This was also in line with the p53 immunohistological finding.

Based on the molecular and clinical findings, the *TP53-*specific ACMG classification of *TP53*(NM_000546.5):c.97-2A>C in this proband is Class 5, “pathogenic,” because it affects a splice site (PVS1_strong), is not found in gnomAD exomes or genomes (PM2_supporting), and matches computational predictions (PP3_mooderate). Furthermore, our additional evidence supports its pathogenicity: i) exon-skipping using an *in vitro* functional test and ii) the negative *TP53* immunohistochemistry on the tumor tissue.

## Discussion

We identified the *TP53*(NM_000546.5):c.97-2A>C variant as a novel, germline pathogenic alteration in the background a thigh-muscle PEComa. This variant, to the best of our knowledge, has not been previously reported in the literature. In the ClinVar database, a single submitter reported a different variant at the same localization (accession: VCV000246337.1; NM_000546.5:c.97-2A>G), but the molecular *in vitro* characterization of this variant has not been performed. Additionally, another variant affecting the same splice site at a different localization, NM_000546.5:c.97-1G>A, was identified in a patient meeting Chompret criteria and was found to cause abnormal splicing upon functional assay analysis (21, 22). We proved that the newly identified NM_000546.5:c.97-2A>C variant led to exon 4 skipping, potentially resulting in a different p53 protein structure that would be predicted to have decreased stability. This is supported by p53 immunohistochemistry, where tumor cells were predominantly negative, and only scattered, focal positivity could be seen in pleomorphic cells.

PEComas (perivascular epithelioid cell tumors) are rare, mesenchymal tumors of uncertain malignant potential, as recurrences may occur years after the initial diagnosis. Malignant metastasizing PEComas are very rare (23). The differential diagnosis can include carcinomas, smooth muscle tumors, and adipocytic neoplasms (23). Our case (first diagnosed as myxofibrosarcoma of the muscle) highlights the difficulties in the pathological diagnosis of malignant PEComa. Regarding PEComa pathogenesis, alterations in two, or recently three, main pathways have been described. Most commonly, a loss of function in the tuberous sclerosis complex subunit 1, *TSC1* (~27%) or *TSC2* (~73%), has been observed due to deletion or pathogenic missense variants, leading to activated mTOR signaling and increased cell growth (23, 24). *TSC1* or *TSC2* inactivation can appear somatically, or in individuals already harboring a germline *TSC1/2* mutation. In both cases, mTOR inhibition can be a potential therapeutic option. The other main molecular feature behind PEComa pathogenesis (in approximately 23% of cases) is the rearrangement affecting *TFE3* (transcription factor binding to IGHM enhancer 3), which is implicated in cell differentiation (23, 24). This has a significant clinical importance, as these tumors might be non-responsive to mTOR inhibition. Lately, rearrangements of *RAD51B* in uterine PEComas have also been identified (23). Similar to other tumors, somatic *TP53* mutations have been described in PEComas, and they are potentially linked to malignancy (24–27).

Most PEComas are sporadic, and only a small subset is associated with the hereditary condition TSC. Recently, our group also identified a *PTCH1* mutation in a patient with bilateral intra-abdominal PEComas suffering from Gorlin-Goltz syndrome (16). PEComas have been reported in only two Li–Fraumeni cases in the literature to date (14, 15). Contrary to these two examples, in our case the PEComa was the first manifestation in the LFS proband. In our case, we could not detect *TSC1* or *TSC2* sequence- or copy-number variants, either in the germline or somatically. The causative role of *RAD51B* was also excluded by multigene panel sequencing, copy number analysis, and fusion analysis. We did not detect *TFE3* rearrangement by FISH analysis, which further reduces the likelihood of a causative role of the *TFE3* pathway in the pathogenesis. However, we identified a site-specific LOH in the PEComa tissue regarding the novel *TP53* pathogenic variant. The normal allele was lost in favor of the non-functional allele harboring the pathogenic variant, and this was supported by the immunohistochemical findings. This suggested a role for the defective *TP53* pathway in the PEComa pathogenesis, which is also reported to be associated with the malignant, metastatic form of this tumor type in this patient.

While a sinonasal carcinoma can be part of the Li–Fraumeni spectrum, we were not able to identify the second hit affecting *TP53* that causes the tumor development.

As *TP53* pathogenic variants contribute to cancer proliferation and metastasis, targeting the signaling pathways that become altered by p53 mutation seems to be an attractive strategy (28). Whereas in the clinical practice there is currently no such drug available, several agents are under investigation in clinical trials (28). The prognostic and predictive role of *TP53* pathogenic variants has been intensively investigated and reported in somatic settings (29). Currently, there are no special recommendations for treatment of the Li–Fraumeni spectrum; indeed, there are reports of treatment (chemo- and radiotherapy) failure (30). While the primary goal is always the treatment of the actual malignant disease, the radiation (both diagnostic and therapeutic) exposure should be minimized, as subsequent primary tumors, particularly within the radiotherapy field, often develop after the exposure (3). Therefore, avoiding radiotherapy when possible and instead using preferably non-genotoxic chemotherapies are recommended by recent guidelines (3).

The genetic counseling of patients carrying pathogenic *TP53* variants is essential. Following international and national guidelines, the patients have to be informed of the disease, the risk of tumor development and localization, the potential options related to surveillance, and the screening of first-degree or at-risk relatives (3, 9). Accordingly, pre- and posttest genetic counseling and family screening were performed in our PEComa patient.

## Conclusion

We identified a novel *TP53* splice variant in an attenuated LFS patient manifesting with a malignant PEComa of unusual appearance. This rare, unexpected phenotype of the patient highlights the importance of the introduction of the Li–Fraumeni spectrum instead of the classic LFS concept. Additionally, using complex molecular genetic assays, we demonstrated the pathogenic role of a novel *TP53* germline variant in the development of the PEComa. This may help with the interpretation of this variant in other patients identified in the future.

## Data Availability Statement

The original datasets presented in this study are available in a publicly accessible repository: NCBI SRA database under accession number PRJNA815946.

## Ethics Statement

The studies involving human participants were reviewed and approved by the Scientific and Research Committee of the Medical Research Council of the Ministry of Health, Hungary (ETT-TUKEB 53720-4/2019/EÜIG). The patients/participants provided their written informed consent to participate in this study. Written informed consent was obtained from the individual(s) for the publication of any potentially identifiable images or data included in this article.

## Author Contributions

Conceptualization, JL, HB, and AP. Germline genetic tests, AB, HB. Regular and molecular pathology tests, MS, ET. Genetic interpretation, HB. Writing—original draft preparation, HB. Writing—review and editing, AP, JL. Visualization, HB. Supervision, AP and JL. Project administration, HB. Funding acquisition, AP and HB. All authors contributed to the article and approved the submitted version.

## Funding

This work was supported by the National Laboratories Excellence program (under the National Tumor biology Laboratory project (NLP-17) and the Hungarian Scientific Research Grant of National Research, Development and Innovation Office (NKFI FK 135065 to HB). HB is a recipient of Bolyai Research Fellowship of the Hungarian Academy of Sciences.

## Conflict of Interest

The authors declare that the research was conducted in the absence of any commercial or financial relationships that could be construed as a potential conflict of interest.

## Publisher’s Note

All claims expressed in this article are solely those of the authors and do not necessarily represent those of their affiliated organizations, or those of the publisher, the editors and the reviewers. Any product that may be evaluated in this article, or claim that may be made by its manufacturer, is not guaranteed or endorsed by the publisher.

## Supplementary Material

Supplementary Table Germline variants detected by exome sequencing (mean allele frequency (MAF) <0.01). All data is available at GeneBank under Submission # 2537264.
